# Transcutaneous electrical acupoint stimulation for preventing postoperative delirium in elderly patients: a systematic review and meta-analysis

**DOI:** 10.3389/fmed.2026.1717702

**Published:** 2026-01-15

**Authors:** Yi-jiao Chen, Qi-hong Shen, Yi Yang, Rui Shen, Hui-fang Li

**Affiliations:** 1 Department of Anesthesiology, Sir Run Run Shaw Hospital Affiliated with the Zhejiang University School of Medicine, Hangzhou, China; 2 Department of Anesthesiology, Affiliated Hospital of Jiaxing University, Jiaxing, China; 3 Department of TCM, Tongxiang Maternal and Child Health-Care Center, Tongxiang, Zhejiang, China

**Keywords:** elderly, meta-analysis, perioperative, postoperative delirium, transcutaneous electrical acupoint stimulation

## Abstract

**Objective:**

To systematically evaluate the efficacy of transcutaneous electrical acupoint stimulation (TEAS) in preventing postoperative delirium (POD) in elderly patients undergoing various surgical procedures.

**Methods:**

A comprehensive literature search was conducted across multiple electronic databases to identify randomized controlled trials (RCTs) comparing TEAS with control interventions (sham or no stimulation) in patients aged >60 years undergoing surgery. The primary outcome was the incidence of POD within the first seven postoperative days. Meta-analysis was performed using RevMan software, calculating risk ratios (RR), mean differences (MD), or standard MD with 95% confidence intervals (CIs). The quality of evidence was assessed based on the Grading of Recommendations Assessment, Development, and Evaluation approach.

**Results:**

Twenty RCTs involving 2,290 patients (aged >60 years) were included. The overall incidence of POD was 5.6% in the TEAS group compared to 17.0% in the control group (RR 0.34, 95% CI 0.26–0.45). TEAS also significantly reduced CAM score (MD −1.01, 95% CI −1.98 to −0.04), propofol consumption (MD −35.59 mg, 95% CI −65.75 to −5.42), postoperative pain score (MD −0.60, 95% CI −1.02 to −0.18), and improved recovery quality (QoR-15 score: MD 23.76, 95% CI 21.72–25.80). The intervention appeared safe with no serious adverse events reported.

**Conclusion:**

Perioperative TEAS application significantly reduces the risk of POD in elderly surgical patients. Its protective effects are potentially mediated through anti-inflammatory effects. TEAS represents a promising non-pharmacological intervention for POD prevention within enhanced recovery protocols.

**Systematic review registration:**

CRD420251128976.

## Introduction

Postoperative delirium (POD) constitutes a prevalent and serious complication in elderly surgical patients, characterized by acute fluctuations in attention, awareness, and cognition (1, 2). Its incidence varies considerably, ranging from 15 to 50% depending on the type of surgery and patient vulnerability, and it is associated with devastating consequences, including prolonged hospitalization, increased healthcare costs, accelerated cognitive decline, higher morbidity and mortality rates, and elevated caregiver burden (3, 4). The aging global population has led to a rising number of elderly patients undergoing surgical interventions, making POD a critical public health concern that imperatively demands effective preventive strategies.

The pathophysiology of POD is multifactorial and not entirely elucidated, but it is widely recognized as a manifestation of cerebral vulnerability precipitated by the stress response to surgery and anesthesia (5). Key mechanistic contributors include neuroinflammation (6), increased permeability of the blood–brain barrier (7), oxidative stress (8), and neuronal injury (9). Current preventive approaches are primarily pharmacological, often involving antipsychotics or other psychoactive medications; however, their efficacy remains limited and their use is frequently associated with significant adverse effects, including sedation, and cardiovascular events (10, 11). This limited risk–benefit profile underscores the urgent need for safe, effective, and non-pharmacological adjunctive interventions.

Transcutaneous electrical acupoint stimulation (TEAS), a non-invasive modality derived from traditional acupuncture principles, applies electrical stimulation to specific acupoints through surface electrodes (12). It has gained increasing attention in perioperative medicine for its potential to modulate physiological functions, including analgesia, anti-emesis, attenuation of the surgical stress response, and organ protection (13–16). Emerging evidence from randomized controlled trials (RCTs) suggests that TEAS may confer protective effects against POD by modulating inflammatory cytokines (17), reducing markers of neuronal injury (18), improving sleep quality (19), and decreasing analgesic requirements (20). However, the findings across individual studies have been inconsistent, and the overall efficacy of TEAS for POD prevention has not been conclusively established due to variations in study design, patient populations, and TEAS protocols.

Therefore, we conducted this systematic review and meta-analysis of RCTs to synthesize the existing evidence and quantitatively evaluate the efficacy of TEAS in preventing POD in elderly patients undergoing surgery. We aimed to evaluate its effect on POD incidence and explore its impact on secondary outcomes including cognitive scores, pain, recovery quality, and key pathophysiological biomarkers.

## Methods

This systematic review and meta-analysis was conducted and reported in accordance with the Preferred Reporting Items for Systematic Reviews and Meta-Analyses (PRISMA) guidelines. The study protocol was registered prospectively on the International Prospective Register of Systematic Reviews (PROSPERO) (Registration number: CRD420251128976).

### Search strategy and data sources

A comprehensive and systematic literature search was performed from inception until July 31, 2025 to identify all relevant published RCTs. The following electronic databases were searched: PubMed, Embase, Cochrane Central Register of Controlled Trials (CENTRAL), Web of Science, China National Knowledge Infrastructure (CNKI), Wanfang Data, and VIP Database. The full detailed search strategy, including all MeSH terms and free-text keywords used for each database, is provided in Supplementary material. In brief, the search concepts included terms related to the intervention (“transcutaneous electrical acupoint stimulation,” “TEAS,” “acupoint”) and the outcome (“postoperative delirium,” “POD,” “delirium”) combined with filters for RCTs and aged population. No language restrictions were applied. The reference lists of all included studies and relevant review articles were also manually screened to identify any additional eligible records.

### Eligibility criteria

Studies were selected based on the following PICOS criteria: *Population (P):* Patients aged ≥60 years undergoing any elective surgical procedure. *Intervention (I):* Perioperative TEAS applied alone or as an adjunct to standard anesthesia care. Studies using any acupoint selection [e.g., Shenmen (HT7), Neiguan (PC6), Hegu (LI4)] and stimulation parameters were included. *Comparison (C):* Sham TEAS (electrodes placed at the same acupoints without electrical stimulation) or standard care/placebo. *Outcomes (O):* The primary outcome was the incidence of POD within the first seven postoperative days, diagnosed using validated tools such as the Confusion Assessment Method (CAM) or its variants. Secondary outcomes included CAM score, intraoperative propofol and remifentanil consumption, postoperative pain score, quality of recovery (QoR-15 score), levels of neuronal injury markers (TNF-*α* and NSE), and other adverse events (e.g., postoperative nausea and vomiting, PONV). *Study Design (S):* Only RCTs were included.

### Study selection and data extraction

Two reviewers independently screened titles, abstracts, and subsequently full-text articles according to the eligibility criteria. Any discrepancies were resolved through discussion or by consultation with a third reviewer. A standardized pre-piloted data extraction form was used to collect information on: first author, publication year, sample size, patient characteristics, surgical type, TEAS protocol (acupoints, parameters, timing), control intervention, and outcomes of interest.

### Risk of bias and quality assessment

The methodological quality of the included studies was assessed using the Cochrane Risk of Bias tool by two independent reviewers. The assessment covered domains of random sequence generation, allocation concealment, blinding of participants and personnel, blinding of outcome assessment, incomplete outcome data, selective reporting, and other potential biases. The overall certainty of evidence for each outcome was evaluated using the Grading of Recommendations, Assessment, Development, and Evaluations (GRADE) approach.

### Data synthesis and statistical analysis

Statistical analysis was performed using Review Manager (RevMan) software (Version 5.4, The Cochrane Collaboration) and Stata software (Version 17.0). For dichotomous outcomes (e.g., POD incidence), the treatment effect was expressed as risk ratios (RR) with 95% confidence intervals (CIs). For continuous outcomes (e.g., pain score, biomarker levels), mean difference (MD) or standardized MD (SMD) were calculated with 95% CIs. Heterogeneity among studies was assessed using the I^2^ statistic and Chi-squared test. An I^2^ value > 50% indicated substantial heterogeneity. Considering multiple factors that contributed to the high clinical heterogeneity in this study, a random-effect model was utilized for the studies with low *I*^2^ values. Pre- specified subgroup analyses were conducted based on surgical type to explore potential sources of heterogeneity. Sensitivity analyses were performed to test the robustness of the results. Publication bias was assessed visually using funnel plots.

## Results

### Study selection

**Figure 1:**
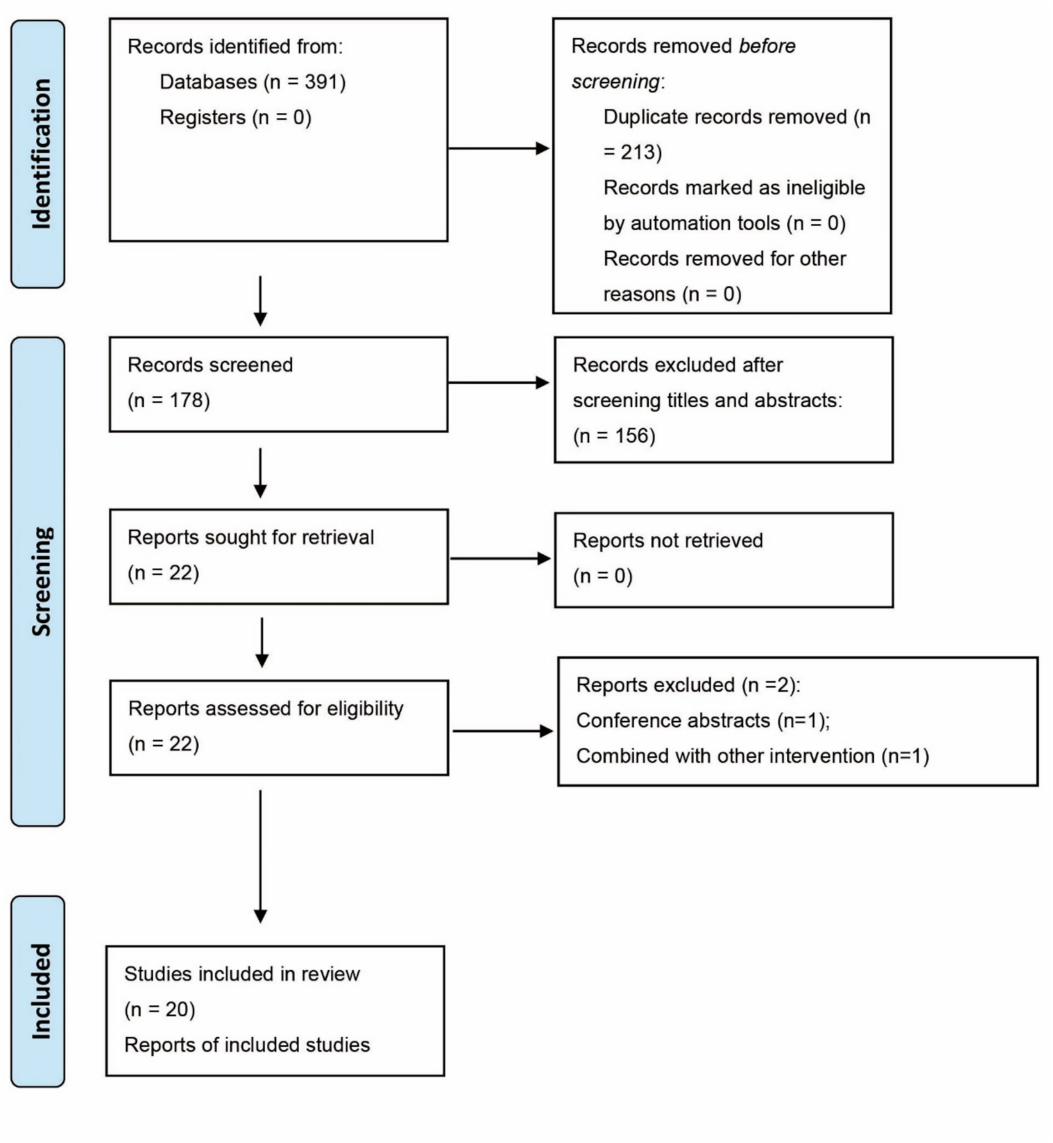
PRISMA flow diagram.

The initial systematic literature search identified a total of 391 records from all electronic databases and other sources. After removing duplicates, 178 unique records remained for title and abstract screening. Following this initial screening, 22 articles were selected for full-text review to assess their eligibility against the pre-defined PICOS criteria. After a detailed evaluation, 20 RCTs met all inclusion criteria and were included in the quantitative synthesis (meta-analysis) (17–36). The detailed process of study selection, with reasons for exclusion at the full-text stage, is presented in the PRISMA flow diagram (Figure 1).

### Study characteristics

The 20 included studies enrolled a total of 2,290 patients, with sample sizes ranging from 57 to 547 participants. All studies involved elderly patients (age >60 years) undergoing various surgical procedures, including but not limited to hip fracture repair, laparoscopic cholecystectomy, radical thyroidectomy, and spinal open surgery. The baseline characteristics of included studies is shown in Table 1. The TEAS interventions utilized common acupoints, predominantly Shenmen (HT7), Neiguan (PC6), Hegu (LI4), and Zusanli (ST36). Stimulation parameters varied across studies, with frequencies often set at 2/100 Hz (dense-disperse mode) and intensity adjusted to patient tolerance (typically 6–15 mA). The control groups received either sham TEAS (placebo stimulation) or standard care. The primary outcome (POD incidence) was assessed using the CAM or its variants in all studies. A detailed summary of the characteristics of the included studies is provided in Table 2.

**Table 1:** Baseline characteristics of included studies.

Study	Age (years)	Sample size	Gender (M/F)	ASA grade	Type of surgery	Duration of surgery	Type of anesthesia
Cao et al. (21)	>60	TEAS: 60 Control: 60	TEAS: 29/31 Control: 32/28	I-II	Total hip replacement arthroplasty	TEAS: 52.8 ± 8.5 min Control: 52.6 ± 8.0 min	Spinal anesthesia
Chang et al. (27)	65–80	TEAS: 44 Control: 41	TEAS: 24/20 Control: 26/15	II-III	Total hip arthroplasty	TEAS: 1.4 ± 0.5 h Control: 1.4 ± 0.4 h	Spinal anesthesia
Ding et al. (18)	>60	TEAS: 28 Control: 29	TEAS: 14/14 Control: 17/12	I-III	Pedicle internal fixation and bone-graft fusion	TEAS: 151 ± 32 min Control: 141 ± 25 min	General anesthesia
Du et al. (29)	66–80	TEAS: 70 Control: 70	TEAS: 36/34 Control: 37/33	I-III	Radical resection of pulmonary carcinoma	TEAS: 162.43 ± 18.82 min Control: 165.65 ± 17.12 min	General anesthesia
Gao et al. (17)	≥65	TEAS: 32 Control: 32	TEAS: 15/17 Control: 18/14	I-II	Spine surgery	TEAS: 136 ± 8 min Control: 134 ± 9 min	General anesthesia
Ge et al. (20)	60–80	TEAS: 37 Control: 39	TEAS: 17/20 Control: 20/19	I-II	Total hip arthroplasty	TEAS: 60.0 ± 10.9 min Control: 57.2 ± 11.2 min	Not mentioned
Liu (31)	65–78	TEAS: 60 Control: 60	TEAS: 42/18 Control: 40/20	II-III	Lobectomy for lung cancer	TEAS: 162.97 ± 18.73 min Control: 165.67 ± 16.82 min	General anesthesia
Lu et al. (14)	≥65	TEAS: 39 Control: 39	TEAS: 8/31 Control: 9/30	II-III	Proximal femoral nail internal fixation	TEAS: 86.0 ± 24.1 min Control: 88.2 ± 30.9 min	Intraspinal anesthesia
Qian et al. (36)	≥65	TEAS: 49 Control: 48	TEAS: 13/36 Control: 15/33	Not mentioned	Hip or knee joint replacement surgery	Not mentioned	General anesthesia or Intraspinal anesthesia
Shi et al. (28)	65–78	TEAS: 32 Control: 34	TEAS: 11/21 Control: 10/24	I-II	Spinal surgery	TEAS: 190.75 ± 37.47 min Control: 181.35 ± 31.36 min	General anesthesia
Wang et al. (22)	>60	TEAS: 88 Control: 86	TEAS: 31/57 Control: 41/45	I-III	Pedicle screw internal fixation	TEAS: 1218.10 ± 45.69 min Control: 214.20 ± 34.10 min	General anesthesia
Wei et al. (35)	≥60	TEAS: 41 Control: 42	TEAS: 6/35 Control: 4/38	I-III	Total knee replacement surgery	Not mentioned	General anesthesia
Wei et al. (24)	65–80	TEAS: 50 Control: 52	TEAS: 17/33 Control: 22/30	II-III	Hip replacement surgery	TEAS: 124 ± 32 min Control: 133 ± 37 min	Combined spinal-epidural anesthesia or general anesthesia
Wen and Liang (32)	≥60	TEAS: 31 Control: 31	TEAS: 5/26 Control: 3/28	I-III	Radical thyroidectomy	Not mentioned	General anesthesia
Wu et al. (26)	65–80	TEAS: 30 Control: 30	TEAS: 18/12 Control: 17/13	II-III	Radical resection of gastrointestinal tumor	TEAS: 146 ± 20 min Control: 149 ± 21 min	General anesthesia
Xu et al. (30)	>60	TEAS: 35 Control: 35	TEAS: 20/15 Control: 13/22	I-III	Single-segment lumbar vertebral surgery	Not mentioned	General anesthesia
Yang et al. (25)	61–85	TEAS: 60 Control: 60	TEAS: 40/20 Control: 39/21	Not mentioned	Hip replacement surgery	Not mentioned	General anesthesia
Yu et al. (33)	65–90	TEAS: 32 Control: 32	TEAS: 12/20 Control: 11/21	I-II	Spinal open surgery	TEAS: 119.2 ± 9.2 min Control: 116.5 ± 6.5 min	General anesthesia
Zhang et al. (19)	65–79	TEAS: 52 Control: 52	TEAS: 27/25 Control: 28/24	Not mentioned	Lower limb surgery	Not mentioned	Subarachnoid block
Zhang et al. (23)	>65	TEAS: 273 Control: 274	TEAS: 107/166 Control: 110/164	I-III	Laparoscopic cholecystectomy	Not mentioned	General anesthesia

Transcutaneous electrical acupoint stimulation, TEAS; American Society of Anesthetists, ASA.

**Table 2:** A detailed summary of the characteristics of the included studies.

Study	TEAS group	Control group	Blinding method	POD assessment	Postoperative analgesia protocol
Cao et al. (21)	Position: PC6, LI4 Timing: from 30 min before the commencement of surgery until 30 min before the end of surgery Parameter: 10 Hz, 7–15 mA	Sham TEAS: electrodes at PC6, LI4, no electrical stimulation	Blinding of Participants: unclear Blinding of Personnel: unclear Blinding of Outcome Assessors: unclear	CAM	PCIA: sufentanil 100 μg
Chang et al. (27)	Position: EX-HN3, PC6, LI4 Timing: 30 min before anesthesia induction until the end of the surgery Parameter: 2/100 Hz, 6–10 mA	Sham TEAS: electrodes at EX-HN3, PC6, LI4, no electrical stimulation	Blinding of Participants: unclear Blinding of Personnel: unclear Blinding of Outcome Assessors: unclear	CAM	PCIA: sufentanil 2.0 μg/kg
Ding et al. (18)	Position: GV20, GV24, PC6, LI4 Timing: 30 min before anesthesia induction until the end of the surgery Parameter: 2/6 Hz, 6–12 mA	Sham TEAS: electrodes at GV20, GV24, PC6, LI4, no electrical stimulation	Blinding of Participants: yes Blinding of Personnel: yes Blinding of Outcome Assessors: yes	CAM	Not mentioned
Du et al. (29)	Position: SP10, ST36, PC6, LI4 Timing: Not mentioned Parameter: 2/100 Hz, 6–10 mA	Sham TEAS: electrodes at SP10, ST36, PC6, LI4, no electrical stimulation	Blinding of Participants: unclear Blinding of Personnel: unclear Blinding of Outcome Assessors: unclear	Not mentioned	Not mentioned
Gao et al. (17)	Position: PC6, LI4 Timing: 30 min before anesthesia induction until the end of the surgery Parameter: 2/100 Hz	Sham TEAS: electrodes at PC6, LI4, no electrical stimulation	Blinding of Participants: unclear Blinding of Personnel: unclear Blinding of Outcome Assessors: yes	CAM-ICU	PCIA: sufentanil 1.5 μg/kg
Ge et al. (20)	Position: SP10, ST36, PC6, LI4 et al. Timing: 30 min before surgery, 2 h, 4 h after surgery, and on the first and second day after surgery, twice a day. Each session lasting for 30 min Parameter: 2/100 Hz	No TEAS: standard care	Blinding of Participants: unclear Blinding of Personnel: yes Blinding of Outcome Assessors: yes	Nursing Delirium Screening Scale	Not mentioned
Liu (31)	Position: LI4, PC6 Timing: from the completion of anesthesia induction until the end of surgery Parameter: 2/100 Hz; 6–10 mA	Sham TEAS: electrodes at LI4, PC6, no electrical stimulation	Blinding of Participants: unclear Blinding of Personnel: unclear Blinding of Outcome Assessors: unclear	CAM–ICU	PCIA: sufentanil 3.0 μg/kg
Lu et al. (14)	Position: PC6, LI4, HT7 Timing: 30 min before anesthesia induction and then once daily on postoperative days 1 to 3, each session lasting for 30 min Parameter: 2/100 Hz, 6–15 mA	Sham TEAS: electrodes at PC6, LI4, HT7, no electrical stimulation	Blinding of Participants: yes Blinding of Personnel: yes Blinding of Outcome Assessors: unclear	Not mentioned	PCIA: sufentanil 10 μg/kg
Qian et al. (36)	Position: LI4, HT7, LR3, SP6 Timing: on the day before the surgery date (AM and PM), intervention on the day of surgery day, and the week after the surgery day, each session lasting for 30 min Parameter: 2/100 Hz	No TEAS: standard care	Blinding of Participants: unclear Blinding of Personnel: unclear Blinding of Outcome Assessors: unclear	CAM	Not mentioned
Shi et al. (28)	Position: GV20, Ex-HN18, HT7, SP6 Timing: 1 day before surgery, 30 min before anesthesia, and 1 day after surgery Parameter: 2/10 Hz; 6–15 mA	Sham TEAS: electrodes at GV20, Ex-HN18, HT7, SP6, no electrical stimulation	Blinding of Participants: unclear Blinding of Personnel: unclear Blinding of Outcome Assessors: unclear	CAM	PCIA: sufentanil 1.0 μg/kg
Wang et al. (22)	Position: LI4, PC6, HT7, GV20 Timing: before anesthesia induction and maintained for 30 min Parameter: 10 Hz	Sham TEAS: electrodes at LI4, PC6, HT7, GV20, no electrical stimulation	Blinding of Participants: yes Blinding of Personnel: yes Blinding of Outcome Assessors: yes	CAM	Not mentioned
Wei et al. (35)	Position: EX-HN3, PC6, LI11, P8, P7, GV20 Timing: 30 min before anesthesia induction until the end of the surgery Parameter: 2/6 Hz, 6–12 mA	Sham TEAS: electrodes at EX-HN3, PC6, LI11, P8, P7, GV20, no electrical stimulation	Blinding of Participants: unclear Blinding of Personnel: unclear Blinding of Outcome Assessors: unclear	CAM	PCIA: dezocine 0.5 mg/kg
Wei et al. (24)	Position: PC6, HT7 Timing: 30 min/session/day, from preoperative day 1 to postoperative day Parameter: 10 Hz	Sham TEAS: electrodes at PC6, HT7, no electrical stimulation	Blinding of Participants: unclear Blinding of Personnel: unclear Blinding of Outcome Assessors: unclear	CAM	PCIA: sufentanil 2.0 μg/kg
Wen and Liang (32)	Position: PC6, LI4, LU7, LU5 Timing: 30 min before anesthesia induction until the end of surgery Parameter: 2/100 Hz	Sham TEAS: electrodes at PC6, LI4, LU7, LU5, no electrical stimulation	Blinding of Participants: unclear Blinding of Personnel: unclear Blinding of Outcome Assessors: unclear	CAM	Not mentioned
Wu et al. (26)	Position: HT7, PC6, ST36 Timing: 30 min before anesthesia induction until the end of the surgery; 30 min at postoperative 24, 48, 72 h Parameter: 2/100 Hz; 1–30 mA	Sham TEAS: electrodes at HT7, PC6, ST36, no electrical stimulation	Blinding of Participants: unclear Blinding of Personnel: unclear Blinding of Outcome Assessors: unclear	CAM	PCIA: sufentanil 1.5–2.0 μg/kg
Xu et al. (30)	Position: LI4, PC6, ST36 Timing: 30 min before anesthesia induction until the end of the surgery Parameter: 30/2 Hz; 6–10 mA	Sham TEAS: electrodes at LI4, PC6, ST36, no electrical stimulation	Blinding of Participants: unclear Blinding of Personnel: unclear Blinding of Outcome Assessors: unclear	Rapid Diagnostic Protocol for Delirium	PCIA: sufentanil 1.8 μg/kg
Yang et al. (25)	Position: LI4, PC6 Timing: 30 min before anesthesia induction Parameter: not mentioned	No TEAS: standard care	Blinding of Participants: unclear Blinding of Personnel: unclear Blinding of Outcome Assessors: unclear	CAM	Not mentioned
Yu et al. (33)	Position: LI4, PC6 Timing: 30 min before anesthesia induction until the end of the surgery Parameter: 2/100 Hz	Sham TEAS: electrodes at LI4, PC6, no electrical stimulation	Blinding of Participants: unclear Blinding of Personnel: unclear Blinding of Outcome Assessors: unclear	CAM	Not mentioned
Zhang et al. (19)	Position: PC6, HT7 Timing: 30 min before surgery and then once daily on postoperative days 1 to 3, each session lasting for 30 min Parameter: 2/100 Hz, 6–15 mA	No TEAS: standard care	Blinding of Participants: unclear Blinding of Personnel: unclear Blinding of Outcome Assessors: unclear	CAM	PCIA: sufentanil 2.0 μg/kg
Zhang et al. (23)	Position: BL32, SP6, CV3, CV4 Timing: 30 min before anesthesia induction and lasted 45 min after surgery Parameter: 4/20 Hz	Sham TEAS: electrodes at BL32, SP6, CV3, CV4, no electrical stimulation	Blinding of Participants: yes Blinding of Personnel: yes Blinding of Outcome Assessors: yes	CAM–ICU	PCIA with sufentanil

Transcutaneous electrical acupoint stimulation, TEAS; Postoperative delirium, POD; Confusion assessment method, CAM; Intensive care unit, ICU; Patient-controlled intravenous analgesia, PCIA; Neiguan, PC6; Hegu, LI4; Yintang, EX-HN3; Zusanli, ST36; Xuehai, SP10; Shenmen, HT7; Tai Chong, LR 3; Baihui, GV20; Lieque, LU7, Chizhe, LU5.

### Risk of bias assessment

**Figure 2:**
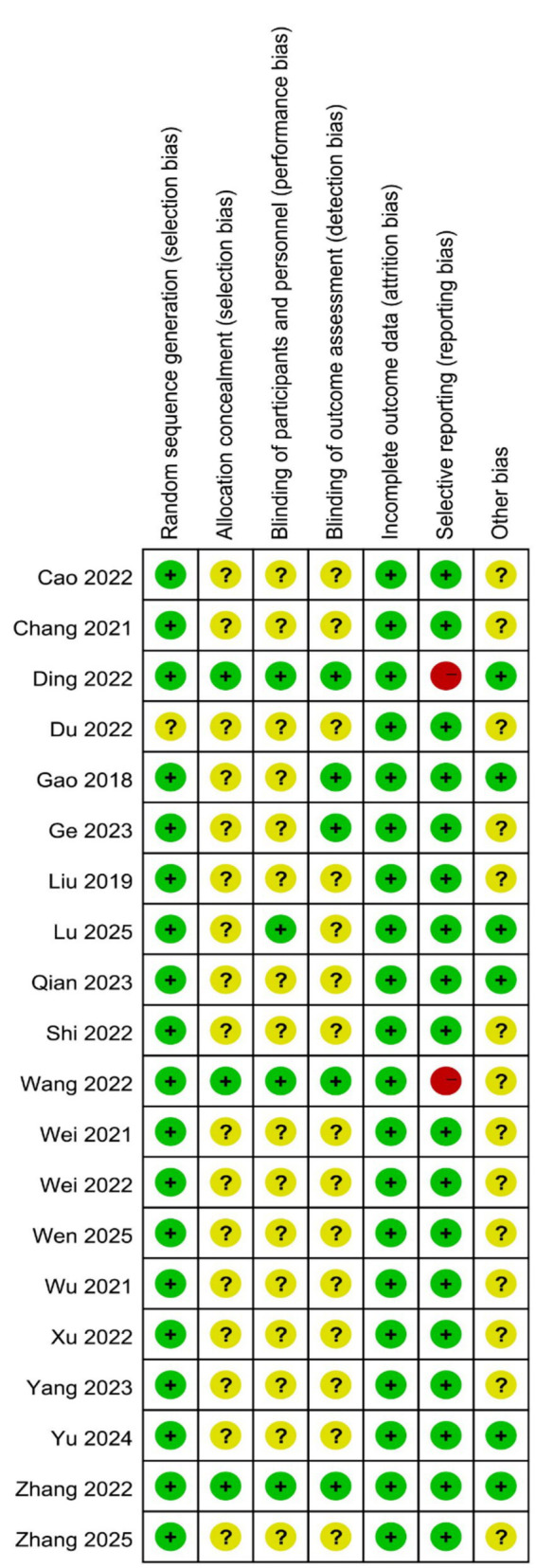
The risk bias assessment of all included studies.

The methodological quality of the included studies was assessed using the Cochrane Risk of Bias tool. Allocation concealment was clearly reported in only three trials. Blinding of participants and personnel was implemented in four trials. Five trials reported blinding of outcome assessment. Two studies had the risk of selective reporting. A visual summary of the risk of bias across all domains is presented in Figure 2.

## Results of meta-analysis

### Primary outcome: incidence of POD

**Figure 3:**
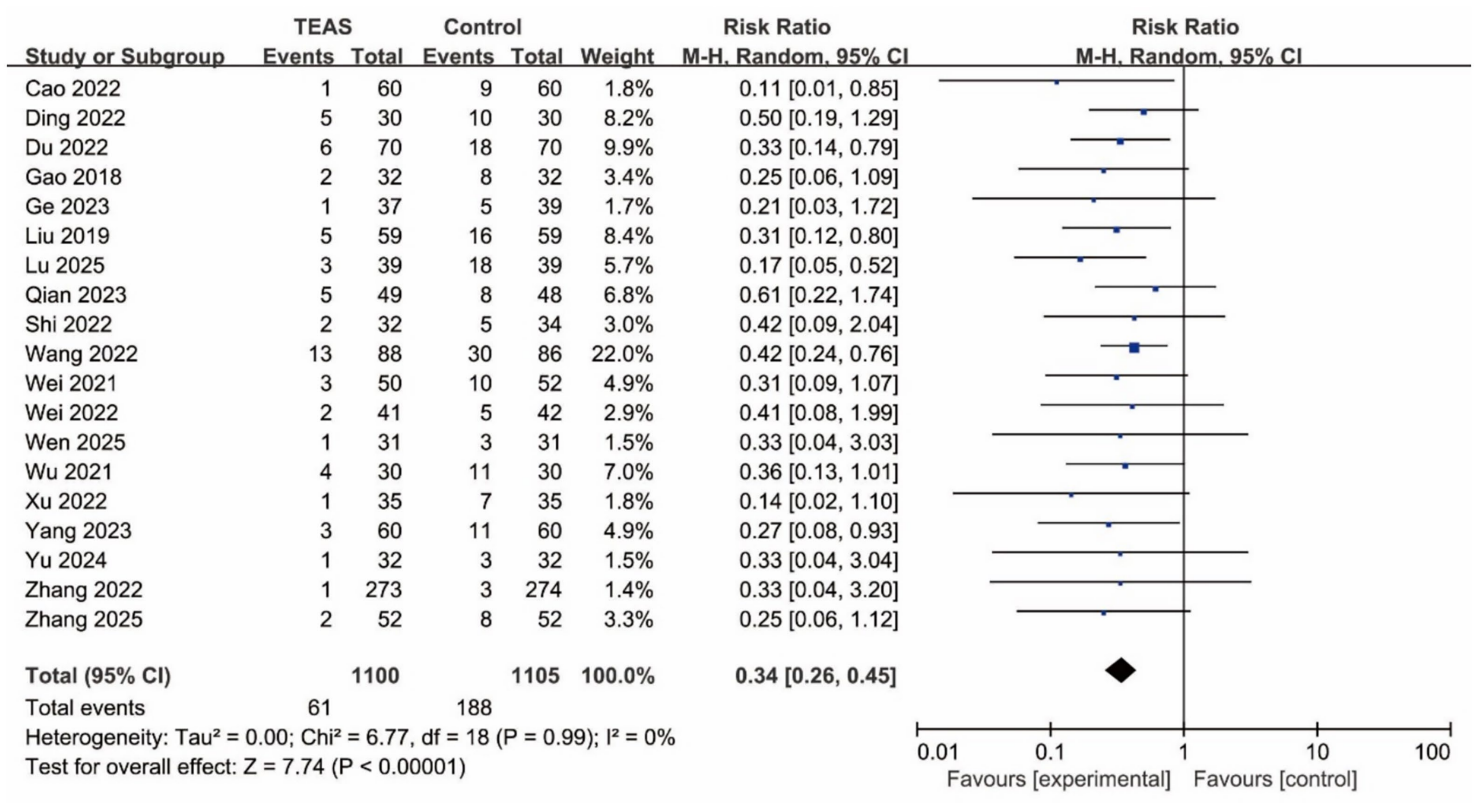
Forest plot of the incidence of POD between TEAS and control group (POD, postoperative delirium; TEAS, transcutaneous electrical acupoint stimulation).

Nineteen studies reported data on the incidence of POD. The pooled meta-analysis using a random-effects model demonstrated that TEAS significantly reduced the risk of developing POD compared to the control group. The overall effect was highly statistically significant (RR = 0.34, 95% CI: 0.26–0.45; *p* < 0.001, *I*^2^ = 0%). The forest plot for the primary outcome is shown in Figure 3.

### Secondary outcomes

**Figure 4:**
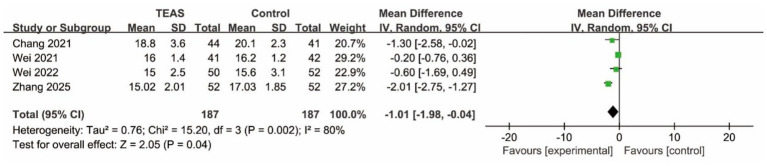
Forest plot of the CAM score between TEAS and control group (CAM, Confusion Assessment Method; TEAS, transcutaneous electrical acupoint stimulation).

*CAM Score:* Pooled analysis from four studies that reported CAM score showed that patients in the TEAS group had significantly lower score than those in the control group (MD = −1.01, 95% CI: −1.98 to −0.04; *p* < 0.05, *I*^2^ = 80%) (Figure 4), indicating less severe delirium symptoms.

**Figure 5:**
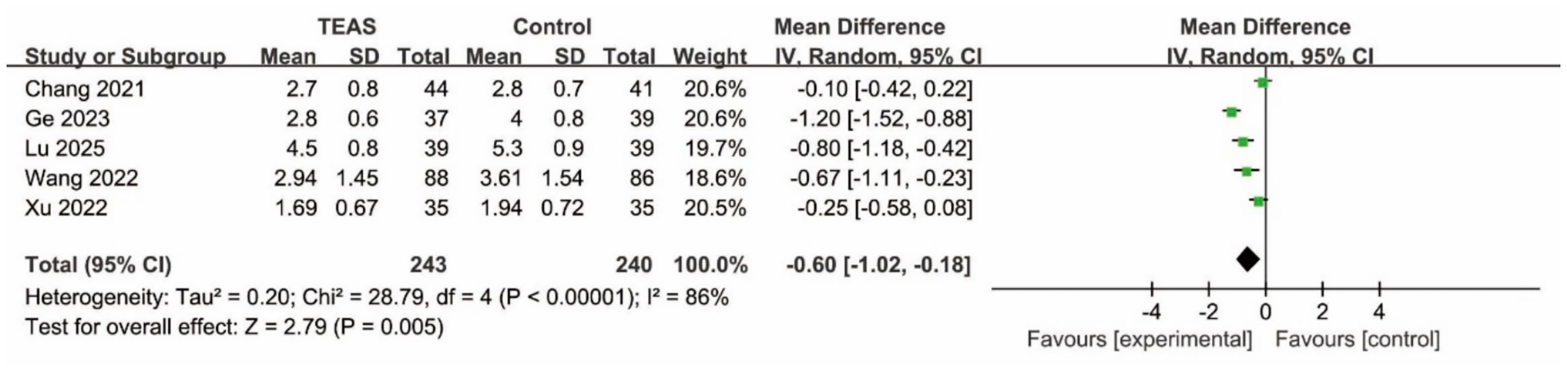
Forest plot of the postoperative pain score between TEAS and control group (TEAS, transcutaneous electrical acupoint stimulation).

*Postoperative Pain Score:* Meta-analysis of five studies assessing pain intensity revealed that TEAS was associated with a statistically significant reduction in postoperative pain score compared to control (MD = −0.60, 95% CI: −1.02 to −0.18; *p* < 0.05, *I*^2^ = 86%) (Figure 5).

**Figure 6:**
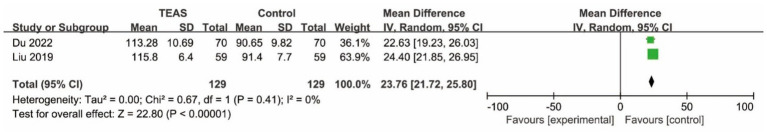
Forest plot of the QoR-15 score between TEAS and control group (TEAS, transcutaneous electrical acupoint stimulation).

*Quality of Recovery:* Data from two studies using the QoR-15 indicated that the TEAS group experienced a significantly better quality of recovery in the postoperative period (MD = 23.76, 95% CI: 21.72–25.80; *p* < 0.05, *I*^2^ = 0%) (Figure 6).

*Inflammatory and Neuronal Injury Biomarkers:* Qualitative synthesis suggested a consistent trend across studies where TEAS was associated with lower postoperative levels of neuronal injury markers (TNF-*α* and NSE) (Supplementary Figures 1, 2) compared to control groups.

**Figure 7:**
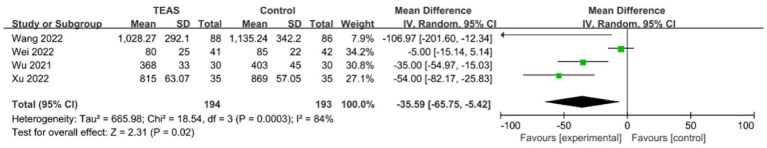
Forest plot of propofol consumption between TEAS and control group (TEAS, transcutaneous electrical acupoint stimulation).

**Figure 8:**
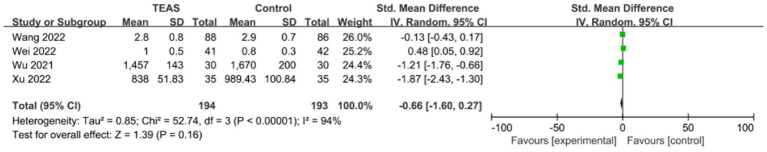
Forest plot of remifentanil consumption between TEAS and control group (TEAS, transcutaneous electrical acupoint stimulation).

*Intraoperative propofol and remifentanil consumption:* The pooled analysis showed a significant reduction in propofol consumption the TEAS group compared to control (MD −35.59 mg, 95% CI −65.75 to −5.42, *p* < 0.05) (Figure 7), while no significant difference in remifentanil consumption (SMD −0.66, 95% CI −1.60–0.27, *p* > 0.05) (Figure 8).

*Adverse Events:* Meta-analysis revealed that no statistically significant difference in incidence of PONV, hypotension and bradycardia between two groups (Supplementary Figures 3–5).

### Subgroup and sensitivity analyses

Pre-specified subgroup analyses based on surgical type (**o**rthopedic surgeries vs. non-orthopedic surgeries) (Supplementary Figure 6) did not identify significant subgroup differences, suggesting a consistent benefit of TEAS across these categories. Sensitivity analyses, performed by excluding each study, did not materially alter the overall pooled estimate for the primary outcome, confirming the robustness of the results.

### Publication bias

**Figure 9:**
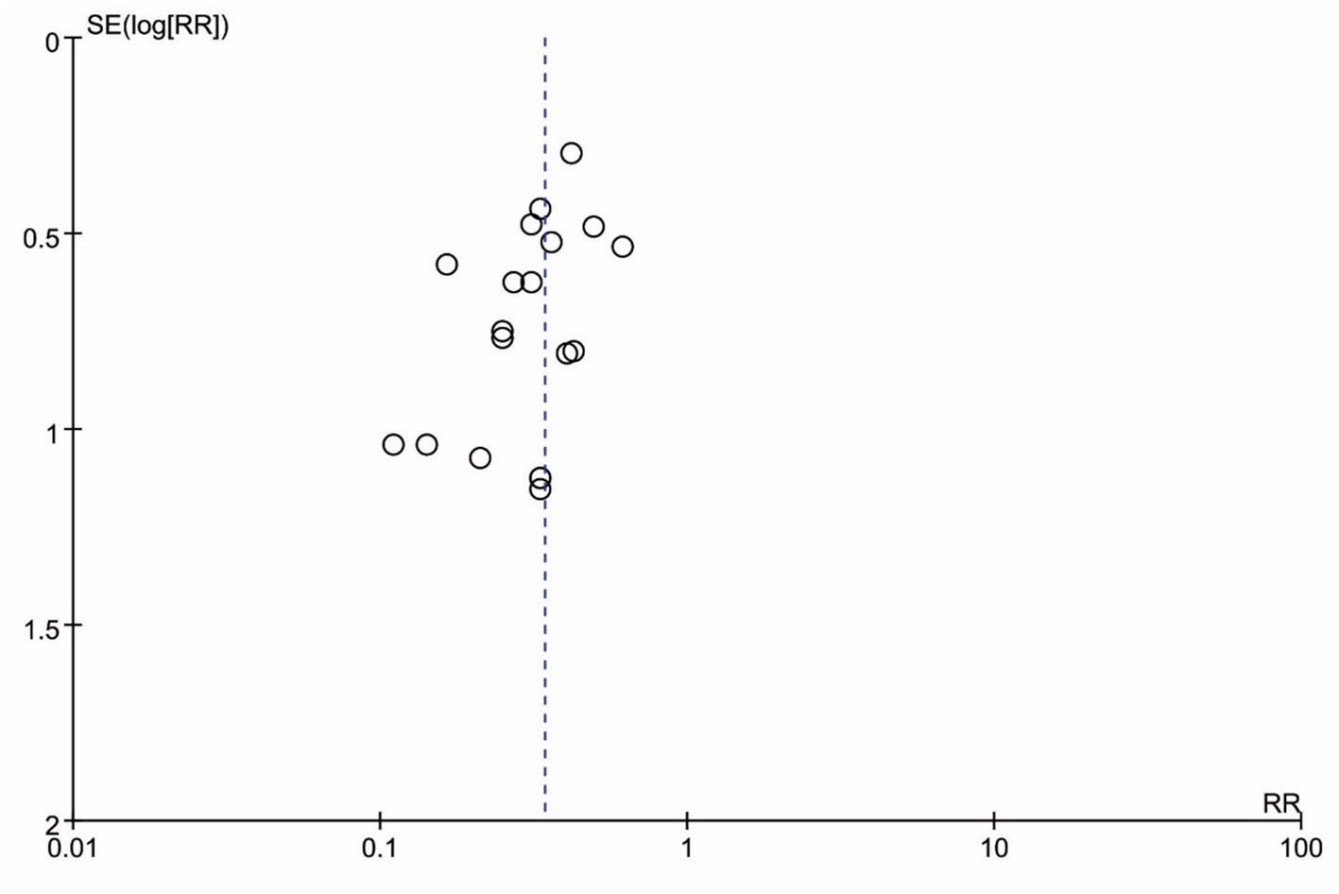
Funnel plot for the primary outcome.

Publication bias was assessed for the primary outcome (incidence of POD) through visual inspection of the funnel plot. The funnel plot displayed an approximately symmetrical distribution of effect estimates around the pooled result (Figure 9). Based on this visual assessment, there was no obvious evidence of significant publication bias among the included studies for this outcome.

### Certainty of evidence (GRADE)

According to the GRADE approach, the certainty of evidence for the primary outcome (POD incidence) was assessed as moderate. The rating “Other considerations” was downgraded “serious.” The certainty of evidence for other outcomes was ranged from low to moderate. Table 3 showed the summary of GRADE.

**Table 3:** Summary for GRADE assessment.

Outcome	Included studies (n)	Patients (n)	RR/MD/SMD	95% CI	I^2^	Quality of evidence	Reasons
Incidence of POD	19	2,205	0.34	(0.26, 0.45)	0%	⨁⨁⨁◯ MODERATE	“Other considerations” was downgraded “serious.”
CAM score	4	374	−1.01	(−1.98, −0.04)	80%	⨁⨁◯◯ LOW	“Imprecision” and “Other considerations” were downgraded to “serious”
Postoperative pain score	5	483	−0.06	(−1.02, −0.18)	86%	⨁⨁◯◯ LOW	“Imprecision” and “Other considerations” were downgraded to “serious”
Quality of recovery	2	258	23.76	(21.72, 25.80)	0%	⨁⨁⨁◯ MODERATE	“Other considerations” was downgraded “serious.”
Propofol consumption	4	387	−35.59	(−65.75, −5.42)	84%	⨁⨁◯◯ LOW	“Imprecision” and “Other considerations” were downgraded to “serious”
Remifentanil consumption	4	387	−0.66	(−1.60, 0.27)	94%	⨁⨁◯◯ LOW	“Imprecision” and “Other considerations” were downgraded to “serious”
TNF-α level	4	418	−14.07	(−25.00, −3.14)	96%	⨁⨁◯◯ LOW	“Imprecision” and “Other considerations” were downgraded to “serious”
NES level	5	592	−3.32	(−4.06, −2.59)	79%	⨁⨁◯◯ LOW	“Imprecision” and “Other considerations” were downgraded to “serious”
Incidence of PONV	4	805	0.36	(0.10, 1.24)	70%	⨁⨁◯◯ LOW	“Imprecision” and “Other considerations” were downgraded to “serious”
Incidence of bradycardia	2	258	0.94	(0.61, 1.45)	0%	⨁⨁⨁◯ MODERATE	“Other considerations” was downgraded “serious.”
Incidence of hypotension	2	258	1.13	(0.68, 1.87)	0%	⨁⨁⨁◯ MODERATE	“Other considerations” was downgraded “serious.”

POD, postoperative delirium; CAM, Confusion Assessment Method; PONV, postoperative nausea and vomiting; RR, risk ratio; MD, mean difference; SMD, standard mean difference; CI, confidence interval.

## Discussion

This systematic review and meta-analysis of 20 randomized controlled trials, encompassing 2,290 elderly surgical patients, provides evidence that perioperative TEAS is associated with a significantly reduced risk of POD. Furthermore, our analysis demonstrates that TEAS confers additional benefits, including amelioration of delirium severity, reduction in postoperative pain, and improvement in the overall quality of recovery. The intervention was found to be safe, with no serious adverse events reported.

The magnitude of the protective effect observed in our study is substantial and underscores the potential of TEAS as a pivotal non-pharmacological strategy within enhanced recovery after surgery (ERAS) protocols (37). The consistency of the beneficial effect across diverse surgical populations, as confirmed by our subgroup analysis showing no significant difference between orthopedic and non-orthopedic surgeries, enhances the generalizability of our findings. This suggests that the neuroprotective mechanism of TEAS may target fundamental pathways common to the pathogenesis of delirium following various surgical insults.

The positive effects of TEAS on secondary outcomes offer critical insights into its potential mechanisms of action. The significant reduction in CAM scores indicates that TEAS not only prevents the onset of delirium but also mitigates its severity. The observed analgesic effect is particularly salient. Postoperative pain is a well- established risk factor for POD; it induces stress, disrupts sleep, and often necessitates increased opioid consumption, which itself can contribute to neuroinflammation and cognitive dysfunction (38, 39). By effectively reducing pain, TEAS may break this vicious cycle, thereby reducing the delirium burden. This is further supported by the marked improvement in the QoR-15 score, indicating a holistic enhancement of the patient’s postoperative experience.

While the exact mechanisms remain to be fully elucidated, our results, combined with the findings of the included studies, point toward multi-modal actions. The qualitative synthesis suggesting a reduction in inflammatory (TNF-*α*) and neuronal injury (NSE) biomarkers aligns with the prevailing neuroinflammatory hypothesis of POD. Surgery triggers a systemic inflammatory response, leading to the release of cytokines that can breach the blood–brain barrier, activate microglia, and disrupt neuronal function and neurotransmission. TEAS has been shown in preclinical and clinical studies to modulate this response, potentially through vagal nerve stimulation and cholinergic anti-inflammatory pathways, leading to a attenuated neuroinflammatory state (40, 41). The underlying pathway may involve the modulation of the cholinergic anti-inflammatory pathway via vagal nerve activation, which suppresses the release of pro-inflammatory cytokines such as TNF-*α* (42). This anti-inflammatory effect, coupled with improved analgesia and sleep quality, likely creates a synergistic effect that protects the vulnerable aging brain from the precipitating factors of delirium.

Our analysis further indicated that TEAS reduced intraoperative propofol requirement while exerting no significant effect on remifentanil consumption. The propofol-sparing effect is clinically meaningful, as propofol may contribute to neuroinflammation in the aging brain (43). TEAS likely provides intrinsic sedation through GABAergic modulation (44), thereby lowering propofol demand and its potential neurotoxic burden.

Several limitations of this analysis must be acknowledged. First, the overall certainty of evidence was graded as moderate for the primary outcome, primarily due to the methodological limitations present in many of the included studies. Common issues included unclear allocation concealment and challenges in blinding personnel administering the intervention, which are inherent difficulties in trials of physical interventions like TEAS. While sham TEAS was employed as control in most studies, the inability to blind personnel may have introduced performance bias. Second, while the funnel plot appeared symmetrical, the possibility of unpublished negative studies (publication bias) can never be completely excluded. Third, we observed substantial statistical heterogeneity (*I*^2^ > 80%) in the meta-analyses for CAM scores and postoperative pain score. This heterogeneity likely stems from clinical diversity, including variations in the specific TEAS protocols (acupoint combinations, stimulation parameters, treatment duration), the types and intensities of surgical procedures, and the different tools used to assess pain. While the random-effects model accounts for this, the findings should be interpreted with awareness of this underlying variability. Fourth, an important clinical dimension of POD, its psychomotor subtype (hyperactive, hypoactive, mixed), could not be analyzed. None of the included studies reported such data. Finally, the majority of included trials were conducted in China, which may limit the generalizability of our findings to other healthcare settings and ethnic populations. Future international multi-center studies are warranted.

## Conclusion

In conclusion, this meta-analysis provides evidence that perioperative TEAS is an effective and safe intervention for preventing postoperative delirium in elderly patients. Its benefits extend beyond mere risk reduction to encompass less severe delirium, improved pain control, and a better overall recovery experience. TEAS might be considered a promising component of multimodal, non-pharmacological strategies aimed at optimizing neurological outcomes and enhancing recovery in the growing population of elderly surgical patients.

## Data Availability

The original contributions presented in the study are included in the article/Supplementary material, further inquiries can be directed to the corresponding author.
