# Supplementary material for: Transcutaneous electrical acupoint stimulation for preventing postoperative delirium in elderly patients: a systematic review and meta-analysis
Source: Front Med (Lausanne). 2026 Jan 15;13:1717702. doi: 10.3389/fmed.2026.1717702 (PMC12852452; doi:10.3389/fmed.2026.1717702)

Search strategies for databases

Pubmed

| Search | Terms |
| --- | --- |
| #1 | "Transcutaneous electrical acupoint stimulation"[Title/Abstract] OR "TEAS"[Title/Abstract] OR "transcutaneous acupoint electrical stimulation"[Title/Abstract] OR "TAES"[Title/Abstract] OR "acustimulation"[Title/Abstract] |
| #2 | ((delirium[Title/Abstract]) OR (postoperative delirium[Title/Abstract])) OR (POD[Title/Abstract]) |
| #3 | ("randomized controlled trial"[Publication Type] OR "controlled clinical trial"[Publication Type] OR "randomized"[Title/Abstract] OR "placebo"[Title/Abstract] OR "clinical trials as topic"[MeSH Terms] OR "randomly"[Title/Abstract] OR "trial"[Title]) NOT ("animals"[MeSH Terms] NOT "humans"[MeSH Terms]) |
| #4 | #1 AND #2 AND #3 |

Web of Science

| Search | Terms |
| --- | --- |
| #1 | TS = (Transcutaneous electrical acupoint stimulation OR TEAS OR transcutaneous acupoint electrical stimulation OR TAES OR acustimulation) |
| #2 | TS = (delirium OR postoperative delirium OR POD) |
| #3 | TS = (randomized OR randomly OR placebo OR trial) |
| #4 | #1 AND #2 AND #3 |

Embase

| Search | Terms |
| --- | --- |
| #1 | 'Transcutaneous electrical acupoint stimulation':ab,ti OR 'TEAS':ab,ti OR 'transcutaneous  acupoint electrical stimulation':ab,ti OR 'TAES':ab,ti OR 'acustimulation':ab,ti |
| #2 | 'delirium':ab,ti OR 'postoperative delirium':ab,ti OR 'POD':ab,ti |
| #3 | ('randomized controlled trial':ab,ti OR ('controlled clinical trial' OR 'randomized':ab,ti OR  'randomly':ab,ti OR 'trial':ab,ti OR 'placebo':ab,ti OR 'clinical article':ab,ti OR 'clinical  trial':ab,ti OR 'controlled study':ab,ti OR 'major clinical study':ab,ti OR 'double blind  procedure':ab,ti OR 'multicenter study':ab,ti OR 'single blind procedure':ab,ti OR 'crossover  procedure':ab,ti)) |
| #4 | #1 AND #2 AND #3 |

The Cochrane Library

| Search | Terms |
| --- | --- |
| #1 | (Transcutaneous electrical acupoint stimulation):ti,ab,kw OR (TEAS):ti,ab,kw OR  (transcutaneous acupoint electrical stimulation):ti,ab,kw OR (TAES):ti,ab,kw OR  (acustimulation):ti,ab,kw |
| #2 | (delirium):ti,ab,kw OR (postoperative delirium):ti,ab,kw OR  (POD):ti,ab,kw |
| #3 | (randomized):ti,ab,kw OR (randomly):ti,ab,kw OR (placebo):ti,ab,kw OR (tiral):ti,ab,kw |
| #4 | #1 AND #2 AND #3 |

China National Knowledge Infrastructure （CNKI）

| Search | Terms |
| --- | --- |
| #1 | (SU = '经皮穴位电刺激' OR SU = '穴位电刺激' OR SU = '经皮电刺激' OR SU = 'TEAS') |
| #2 | (SU = '谵妄' OR SU = '术后谵妄' OR SU = 'POD') |
| #3 | (SU=随机 OR FT=随机) |
| #4 | #1 AND #2 AND #3 |

Wanfang Database

| Search | Terms |
| --- | --- |
| #1 | (经皮穴位电刺激OR穴位电刺激OR经皮电刺激OR TEAS) |
| #2 | (谵妄OR术后谵妄OR POD) |
| #3 | (随机 OR 试验) |
| #4 | #1 AND #2 AND #3 |

Supplement Figure 1. Forest plot of TNF-α between TEAS and control group. (TEAS, transcutaneous electrical acupoint stimulation)


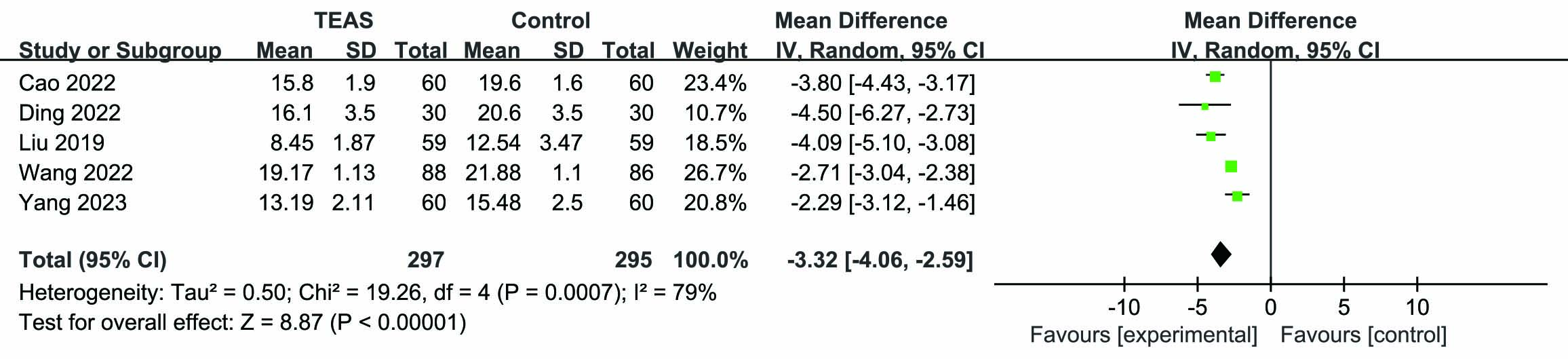


Supplement Figure 2. Forest plot of NES between TEAS and control group. (TEAS, transcutaneous electrical acupoint stimulation)


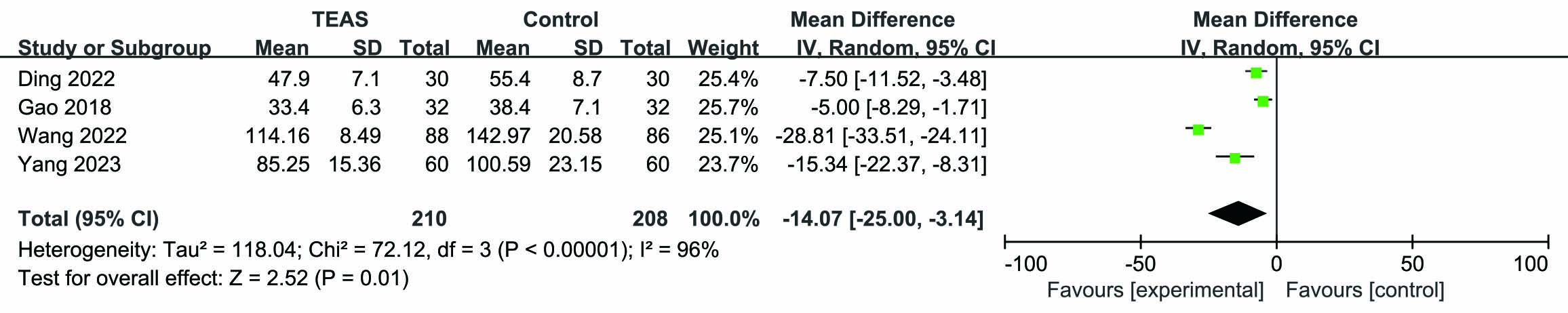


Supplement Figure 3. Forest plot of the incidence of PONV between TEAS and control group. (PONV, postoperative nausea and vomiting; TEAS, transcutaneous electrical acupoint stimulation)


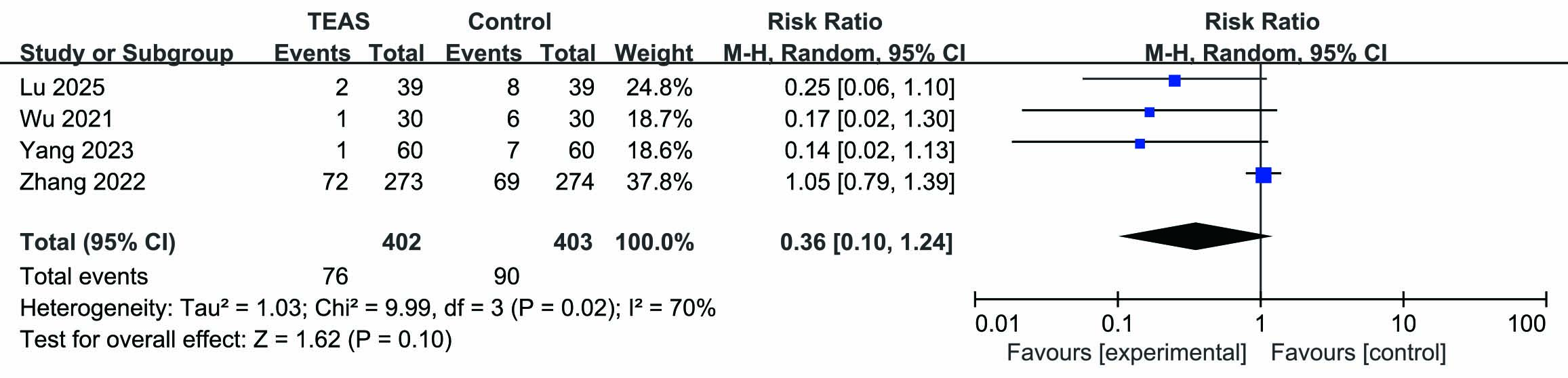


Supplement Figure 4. Forest plot of the incidence of bradycardia between TEAS and control group. (TEAS, transcutaneous electrical acupoint stimulation)


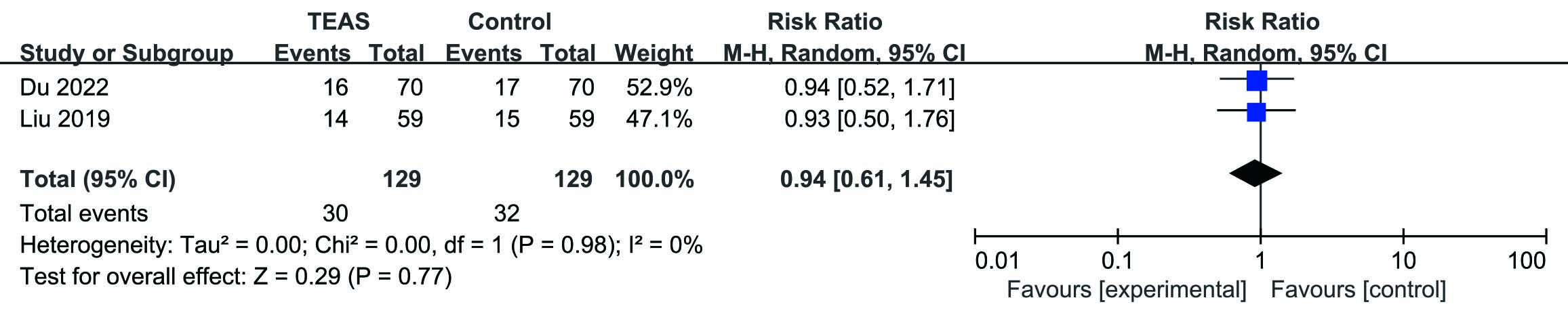


Supplement Figure 5. Forest plot of the incidence of hypotension between TEAS and control group. (TEAS, transcutaneous electrical acupoint stimulation)


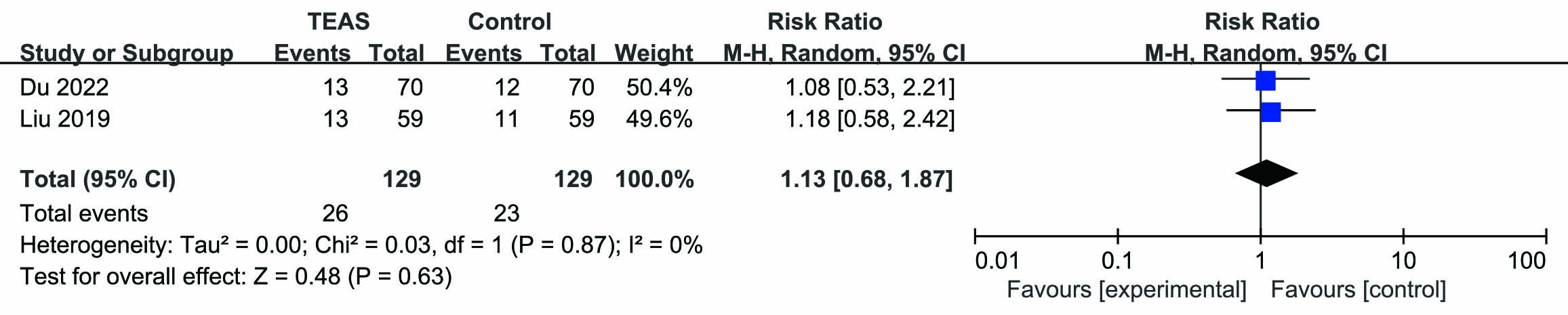


Supplement Figure 6. Subgroup analyses based on surgical type (**o**rthopedic surgeries vs. non-orthopedic surgeries) for the primary outcome.


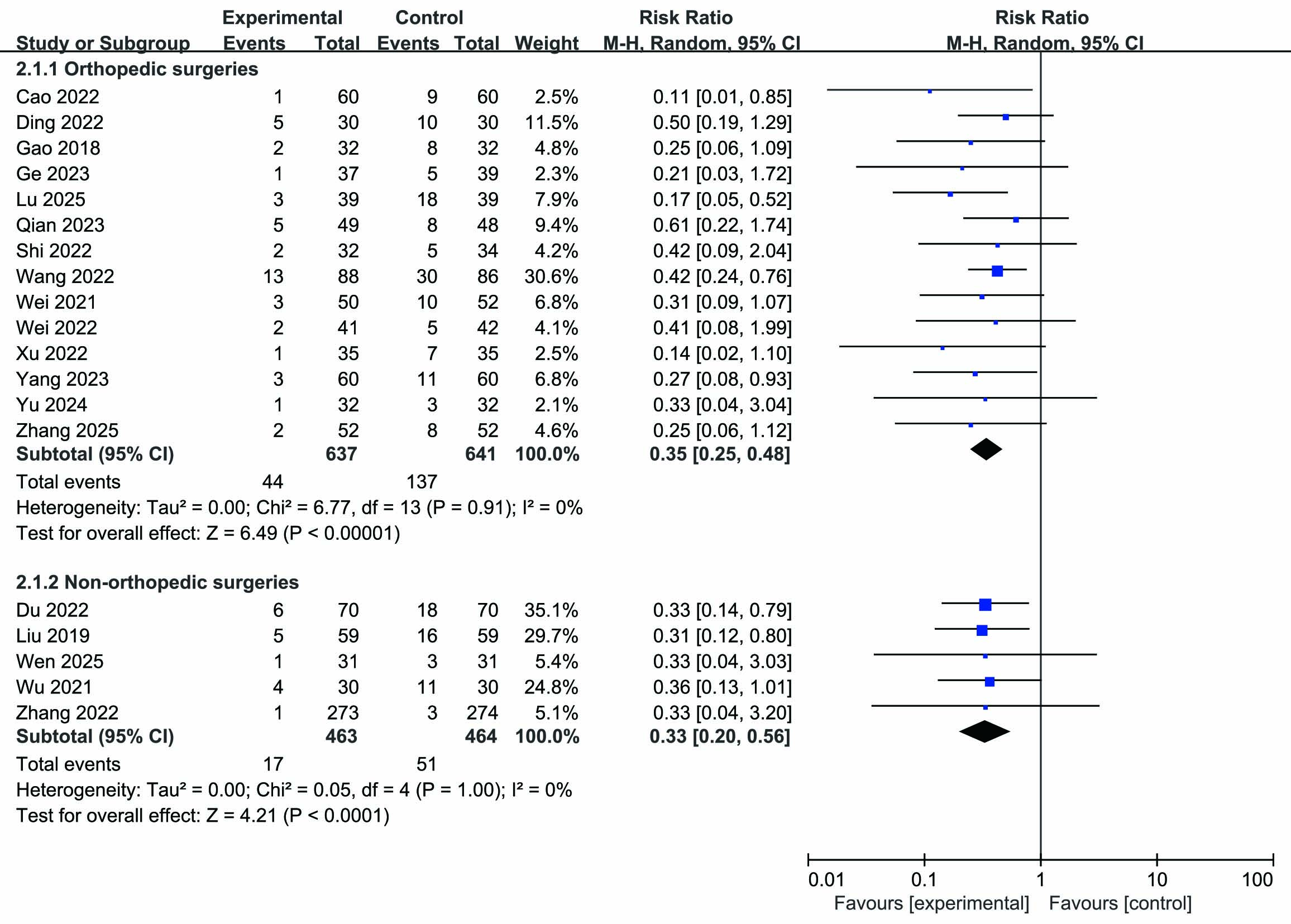

Supplement: Supplementary file 1 [file Table_1.DOC]
